# Different Storage Conditions Influence Biocompatibility and Physicochemical Properties of Iron Oxide Nanoparticles

**DOI:** 10.3390/ijms16059368

**Published:** 2015-04-24

**Authors:** Jan Zaloga, Christina Janko, Rohit Agarwal, Johannes Nowak, Robert Müller, Aldo R. Boccaccini, Geoffrey Lee, Stefan Odenbach, Stefan Lyer, Christoph Alexiou

**Affiliations:** 1 Department of Otorhinolaryngology, Head and Neck Surgery, Section for Experimental Oncology and Nanomedicine (SEON), Else Kröner-Fresenius-Stiftung-Professorship, University Hospital Erlangen, Erlangen 91054, Germany; E-Mails: jan.zaloga@uk-erlangen.de (J.Z.); christina.janko@uk-erlangen.de (C.J.); rohitag.jpr@gmail.com (R.A.); stefan.lyer@uk-erlangen.de (S.L.); 2 Chair of Magnetofluiddynamics, Measuring and Automation Technology, Technische Universität Dresden, Dresden 01062, Germany; E-Mails: johannes.nowak@tu-dresden.de (J.N.); stefan.odenbach@tu-dresden.de (S.O.); 3 Leibniz Institute of Photonic Technology, Jena 07745, Germany; E-Mail: robert.mueller@ipht-jena.de; 4 Institute of Biomaterials, Department of Materials Science and Engineering, University of Erlangen-Nuremberg, Erlangen 91058, Germany; E-Mail: aldo.boccaccini@ww.uni-erlangen.de; 5 Division of Pharmaceutics, University of Erlangen-Nuremberg, Erlangen 91058, Germany; E-Mail: geoff.lee@fau.de

**Keywords:** magnetic drug targeting, iron oxide nanoparticles, nanomedicine, colloidal stability, nanoparticle stability, iron oxide biocompatibility, magnetite maghemite biocompatibility

## Abstract

Superparamagnetic iron oxide nanoparticles (SPIONs) have attracted increasing attention in many biomedical fields. In magnetic drug targeting SPIONs are injected into a tumour supplying artery and accumulated inside the tumour with a magnet. The effectiveness of this therapy is thus dependent on magnetic properties, stability and biocompatibility of the particles. A good knowledge of the effect of storage conditions on those parameters is of utmost importance for the translation of the therapy concept into the clinic and for reproducibility in preclinical studies. Here, core shell SPIONs with a hybrid coating consisting of lauric acid and albumin were stored at different temperatures from 4 to 45 °C over twelve weeks and periodically tested for their physicochemical properties over time. Surprisingly, even at the highest storage temperature we did not observe denaturation of the protein or colloidal instability. However, the saturation magnetisation decreased by maximally 28.8% with clear correlation to time and storage temperature. Furthermore, the biocompatibility was clearly affected, as cellular uptake of the SPIONs into human T-lymphoma cells was crucially dependent on the storage conditions. Taken together, the results show that the particle properties undergo significant changes over time depending on the way they are stored.

## 1. Introduction

Superparamagnetic iron oxide nanoparticles (SPIONs) have attracted increasing attention in many biomedical fields during the past decades. SPIONs consist of cores of magnetic iron oxides, namely magnetite (Fe_3_O_4_), maghemite (γ-Fe_2_O_3_) or a mixture of these. Their feasibility for applications like magnetic drug targeting (MDT), hyperthermia or imaging has been frequently demonstrated [1,2,3,4,5]. At the interface of physical and chemical synthesis and characterisations and biological application, many questions are still to be clarified. In magnetic drug targeting, SPIONs are injected into a tumour supplying artery and accumulated locally in the tumour by a constant magnetic field [6]. This greatly enhances bioavailability of drugs and reduces side effects. However, the effectiveness of this therapy is dependent on magnetic properties, colloidal stability and biocompatibility of the particles [7]. Handling and storage of particles can influence the properties of nanoparticles greatly due to their high surface to volume ratio [8,9]. A good knowledge of the nature of the effects of storage conditions on product stability, physicochemical properties and biocompatibility is of utmost importance for the translation of any therapy concept into the clinic. This is already the case at the research and development stage, as the reproducibility of biological experiments can also be affected by any potential change in the physicochemical properties of SPIONs.

One of the potential effects on lyophobic colloids is the formation of clusters and precipitates, which is unwanted in biomedical applications [10]. Furthermore, colloidal instabilities of SPIONs depending on the storage conditions have been reported before [11] and therefore the storage conditions need to be chosen appropriately to prevent this effect. Little is known about temperature-related processes during mid and long-term storage and their relation to biocompatibility, cellular uptake and physicochemical properties of SPIONs. It has been frequently reported that size and surface charge of nanoparticles greatly influence their effect on biological systems [12,13]. Other studies suggested that oxidation of magnetite to maghemite may influence the biocompatibility of iron oxide particles [14,15]. However, since the hydrodynamic sizes and surface charges of these different particle types in these studies are not always equal it can be difficult to discriminate the influence of the oxidation state of the cores as this effect is possibly superimposed by other effects. Oxidation of the iron oxide cores, however, could also affect the magnetic properties of the SPIONs, as maghemite (γ-Fe_2_O_3_) has a lower saturation magnetization than magnetite (Fe_3_O_4_) [16]. For high efficiency of magnetic drug targeting a high saturation magnetisation of the solution is essential [17]. As Ostwald ripening of the individual iron oxide cores may also significantly influence particle characteristics like their magnetic properties [18], it is important to investigate if the iron oxide core size is changing over time.

In this study, hybrid coated particles with a core-shell structure consisting of multiple iron oxide cores coated with lauric acid and a shell consisting of albumin (SEON^LA–BSA^) were synthesised and stored at different temperatures from 4 to 45 °C over the course of twelve weeks. The particles were tested periodically in order to detect any potential changes over time. In case of SEON^LA–BSA^ the outer surface layer consists of a protein, which has been reported earlier to be sensitive to conformational changes by enhanced temperatures even after short exposure [19]. As enhanced temperatures can also affect the interaction of proteins with nanoparticles [20], it is therefore important to monitor chemical and colloidal stability of such particles during storage at different temperatures. We employed photon cross-correlation spectroscopy (PCCS) and dynamic light scattering (DLS) to monitor hydrodynamic size and surface charge of the particles. The zeta potential, which determines the electrokinetic mobility of particles in a field gradient, can also provide information about the electrostatic repulsion between particles and is therefore related to particle stability. By fourier transform infrared spectroscopy (FT-IR) the particles were examined for any chemical changes in the shell structure. Size and shape of the individual iron oxide cores were examined at the end of the study using transmission electron microscopy (TEM). We performed flow cytometry experiments on human T-lymphoma cells every two weeks in order to investigate the biocompatibility of SEON^LA–BSA^. Thus we investigated cellular uptake, phosphatidylserine exposure, plasma membrane integrity and mitochondrial membrane potential as well as DNA degradation in order to obtain detailed information about potential changes due to storage. Using vibrating sample magnetometry (VSM), we periodically examined the saturation of the magnetisation of SEON^LA–BSA^ at high field strengths in order to monitor any potential time-dependent effects. We used X-ray diffraction (XRD) in order to detect any temperature-dependent changes in the structure of the iron oxide cores over time.

By this study we intend to provide primary information about the effects of storage of SPIONs on their physicochemical properties and biocompatibility.

## 2. Results and Discussion

Four hundred mL of SEON^LA–BSA^ particles were synthesized as one batch as previously described [21]. The total iron content of this suspension was determined as 2.71 ± 0.051 mg/mL total iron.

The sterilised suspension was portioned into glass vials and stored at 4, 20, 37 and 45 °C, respectively. Over the course of twelve weeks, the particles were characterised for their physicochemical properties and *in vitro* biocompatibility.

### 2.1. Storage Temperature Affects Physicochemical Properties of Lauric Acid/Albumin Coated Iron Oxide Nanoparticles (SEON^LA–BSA^)

**Figure 1:**
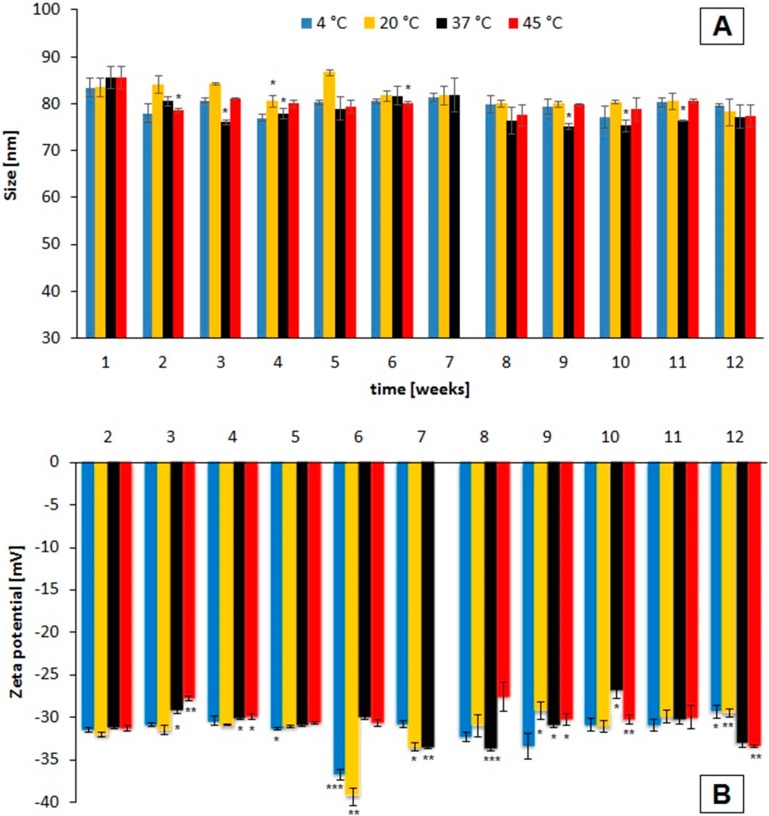
Hydrodynamic diameter (**A**) and zeta potential (**B**) of SEON^LA–BSA^ after different storage times depending on the temperature. The hydrodynamic diameter is given as volume mean diameter. All values are displayed as mean ± standard deviation. All measurements were performed in triplicates. The asterisks mark significant differences in comparison to the original values. The significance levels were determined using a paired *t*-test (* *p* < 0.05; ** *p* < 0.005; *** *p* < 0.0005).

Figure 1 displays the changes in hydrodynamic size and zeta potential of the SPIONs over time depending on storage conditions. The particles were measured weekly under the same conditions by dynamic light scattering (DLS) and photon cross-correlation spectroscopy (PCCS). Although some significant differences in hydrodynamic size and zeta potential were detected, no clear overall trend was visible. The hydrodynamic diameters of SEON^LA–BSA^ which were measured at week twelve did not significantly differ from the ones measured at week one (*p* > 0.05).

**Figure 2:**
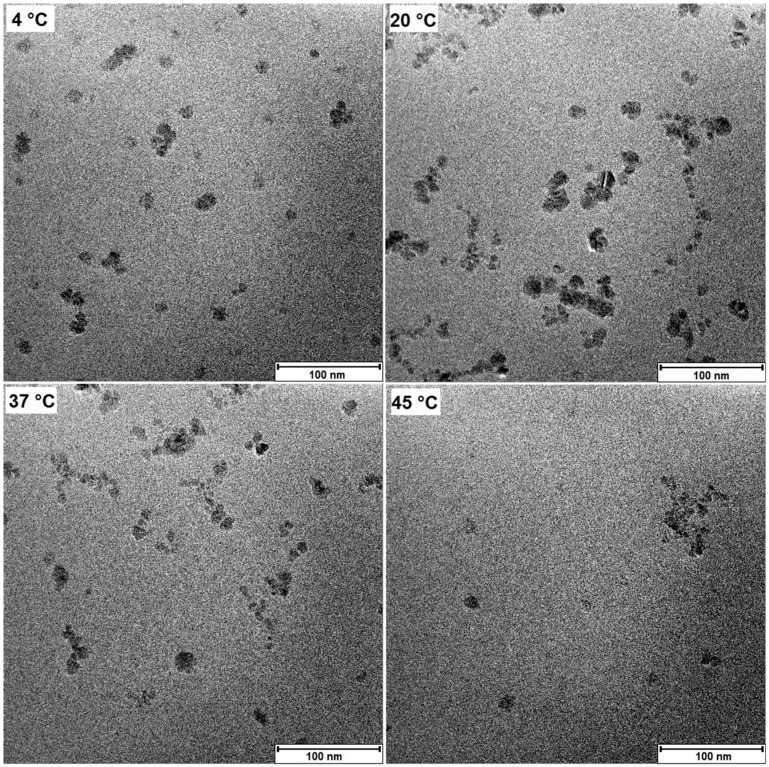
Transmission electron microscopy (TEM) images after storage. Exemplary transmission electron microscopy images after 12 weeks of storage at different temperatures. The individual particles form clusters which are all located inside an organic matrix.

Using transmission electron microscopy (TEM) we investigated the influence of the storage conditions size of the individual iron oxide cores by calculating the sizes from the images seen in Figure 2. The calculated core sizes do not significantly differ (*p* > 0.05) from each other after 12 weeks of storage (see Table 1).

**Table 1:** Calculation of the iron oxide core sizes of particles from the TEM images taken at week 12. The number of individual particles counted was at least *n* = 62 for all ferrofluids.

Storage Temperature (°C)	4 °C (*n* = 72)	20 °C (*n* = 78)	37 °C (*n* = 62)	45 °C (*n* = 72)
Core diameter (nm)	8.9 ± 2.2	8.9 ± 1.9	9.4 ± 1.7	9.2 ± 2.3

**Figure 3:**
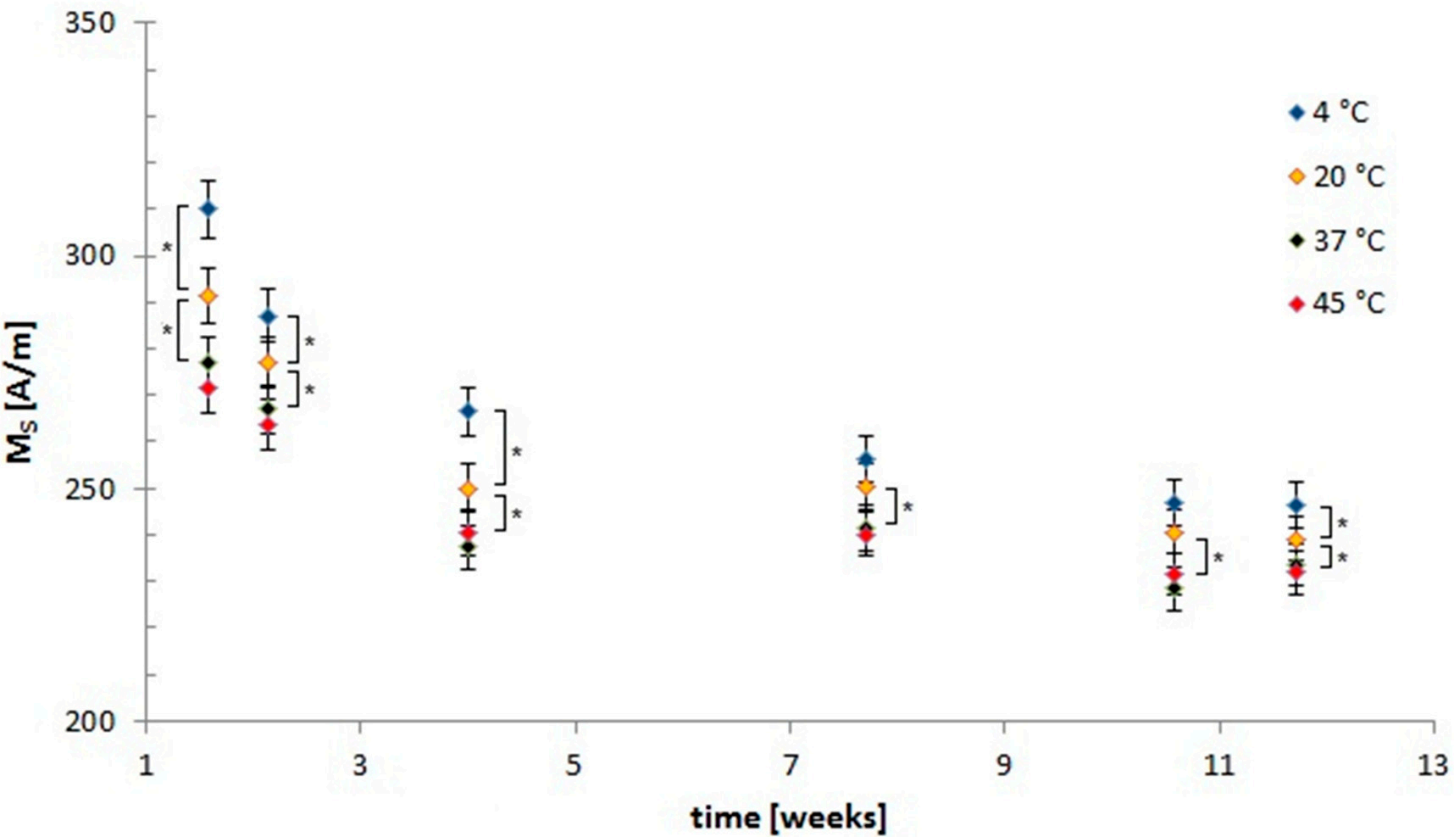
Changes in saturation magnetisations over time. Saturation magnetisation (M_s_) of SEON^LA–BSA^ suspensions after storage at different temperatures are given depending on the storage time. The time points are given as weeks after synthesis. Values are displayed as mean ± standard deviation. All measurements were performed in triplicates. The asterisks mark significance levels that were determined using a paired *t*-test (* *p* < 0.05).

We measured the saturation magnetization (M_S_) of SEON^LA–BSA^ using vibrating sample magnetometry (VSM). In Figure 3 the changes in M_S_ over time are displayed. The decrease in saturation magnetisation is greatly dependent on time and storage temperature, with the main effects occurring within the first four weeks. When SEON^LA–BSA^ is stored at 45 °C, M_S_ decreases significantly (*p* < 0.0005) from 310.1 ± 6.2 to 240.4 ± 4.8 A/m after that time. This equals a decrease by 28.9%. After twelve weeks of storage M_S_ is significantly (*p* < 0.0005) reduced by 33.7% compared to M_S_ of SEON^LA–BSA^ stored at 4 °C for one week. This time-dependent decrease is not prevented, but slowed during storage at 4 °C, as M_S_ decreases by only 16.4% during the first four weeks in this case. The differences between the respective highest and lowest saturation magnetization as a function of storage temperature M_S_(T) decrease from 17.5% at week one to 6.22% at week twelve. These differences were still significant between the 4 °C and the 20 °C and the 20 °C and the 37 °C sample at week twelve. The differences in M_S_(T) between the 37 °C and the 45 °C samples were not significant at any temperature.

**Figure 4:**
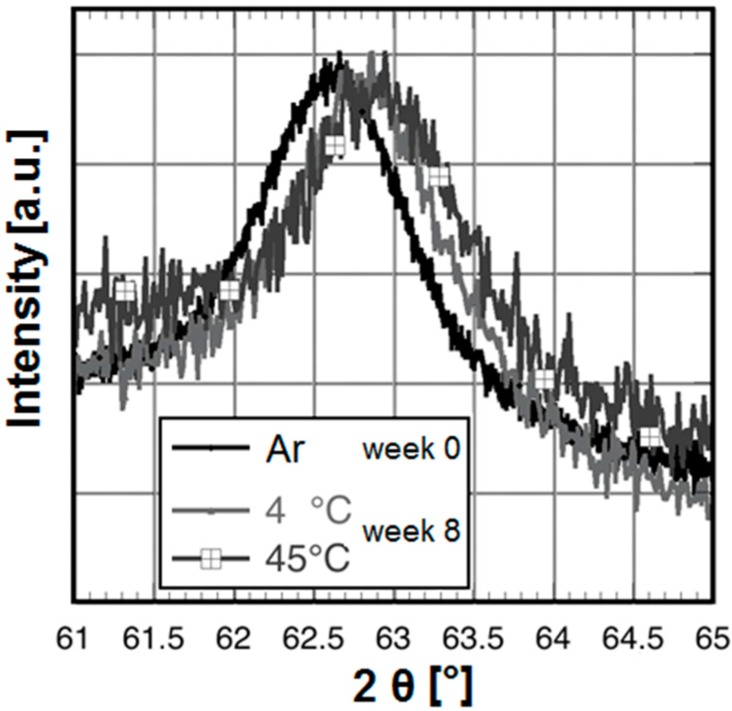
X-ray spectra of particles directly after synthesis and after storage. X-ray diffraction (XRD) spectra of the 440 peak of SEON^LA–BSA^ directly after synthesis (handled under Argon atmosphere until the measurement), and after eight weeks (4 and 45 °C particles).

To investigate whether any potential chemical changes in the molecular structure of the particle system had occurred, we performed FT-IR spectroscopy routinely. However, even after twelve weeks no differences were observed in the wavenumbers or intensity ratios of the peaks (data not shown). The suspension, however, underwent a visible change in colour over time, as it went from black–brown (4 °C samples) to a lighter, reddish–brown (37 and 45 °C) colour. To investigate the origin of this effect we analysed the crystal structure of the iron oxide cores using X-ray diffraction (XRD) after eight weeks of storage and directly after synthesis. With increasing storage time and temperature a small shift of the 440 peak position to higher 2θ angles can be observed, corresponding to an oxidation from Fe_3_O_4_ (JCPDS No 19-0692, 440 peak maximum at ≈62.52°) to γ-Fe_2_O_3_ (JCPDS No 39-1346, 440 peak maximum at ≈62.92°). Figure 4 shows the positions of the 440 peaks of the samples directly after synthesis and at week eight at 4 and 45 °C, respectively.

Despite the stoichiometric Fe^2+^/Fe^3+^ ratio and Ar atmosphere during preparation the sample week 0 is already slightly oxidized in comparison to bulk Fe_3_O_4_. It seems that further oxidation depends more strongly on time than on the storage temperature. Samples of week eight, especially the one stored at 45 °C indicate mostly γ-Fe_2_O_3_ instead of a Fe_3_O_4_/γ-Fe_2_O_3_ solid solution. There is no formation of haematite (α-Fe_2_O_3_). The 45 °C-sample reveals a very small non-identified peak at about 38° that does not belong to spinel or haematite.

It can be concluded that storing the particles for twelve weeks did not affect their colloidal stability. Surprisingly, storage did not affect the protein shell at the chosen experimental conditions and thus had no significant influence on zeta potential or hydrodynamic cluster size. The saturation magnetisation, however, decreased by up to 28.8% after twelve weeks depending on the storage temperature. This effect goes along with a change in suspension colour. This is most likely related to oxidation of the iron oxide cores, as was shown by X-ray diffraction.

### 2.2. Biocompatibility Is Affected Similarly during Storage

The biocompatibility of SEON^LA–BSA^ directly after synthesis has been shown before in a broad concentration range [21]. In order to investigate the effect of storage conditions on cellular interaction and biocompatibility of the SEON^LA–BSA^ particles, different concentrations of SEON^LA–BSA^ ranging from 50 to 300 µg/mL total iron were tested on Jurkat cells. Untreated cells served as controls. We performed flow cytometry experiments every two weeks, investigating cellular morphology, phosphatidylserine exposure, plasma membrane integrity, mitochondrial membrane potential and DNA degradation of the cells after 24 and 48 h of incubation with the respective particles.

**Figure 5:**
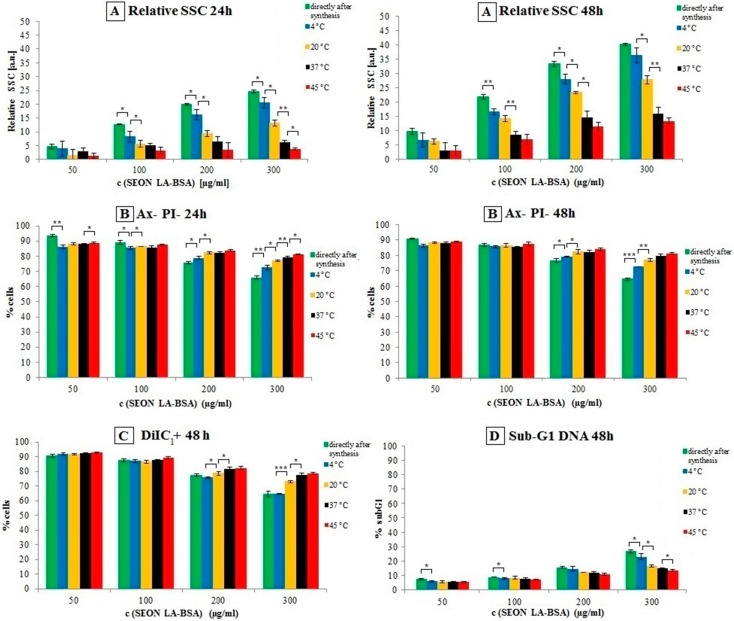
Comparison of biocompatibility of SEON^LA–BSA^ directly after synthesis (green bars), and after four weeks of storage at 4 °C (blue bars), 20 °C (yellow bars), 37 °C (black bars) and 45 °C (red bars). (**A**) Increase of the relative side scatter (SSC) in viable cells after 24 and 48 h of incubation. The relative SSC is given as relative to the respective untreated controls; (**B**) Percentage of cells which were negative for both Annexin A5-Fluorescein isothiocyanate (Ax) and propidium iodide (PI) staining after 24 and 48 h of incubation; (**C**) Percentage of DiIC_1_ positive cells after 48 h of incubation; (**D**) Percentage of cells with sub-G1 DNA after 48 h of incubation. All experiments were performed in triplicates. The asterisks mark significance levels that were determined using an unpaired *t*-test (* *p* < 0.05; ** *p* < 0.005; *** *p* < 0.0005).

Figure 5 shows measurements of cells treated with SEON^LA^^–BSA^ directly after synthesis (week 0) and after four weeks of storage at various temperatures. The particles exhibited an incubation time and concentration-dependent effect on Jurkat cells for all investigated parameters. The side scatter (SSC) reflecting the granularity of cells is an indicator for nanoparticle uptake [22]. In this case, it was increasing in a concentration-dependent manner for all four particle samples (Figure 5A). Interestingly the SSC increase was reduced significantly over time by increasing storage temperatures. By AnnexinA5 (Ax)/propidium iodide (PI) we discriminated viable (negative for Ax and PI), apoptotic (positive for Ax, negative for PI) and necrotic (positive for both stainings) cells. Ax binds to phosphatidylserine, which is exposed on apoptotic plasma membranes. After plasma membrane rupture during necrosis, the normally impermeable dye PI can enter the cells and intercalate into the DNA [23]. Interestingly, the percentage of Ax and PI negative cells, reflecting the number of viable cells, was only affected at very high concentrations of 200 µg/mL total iron and above. As the particle uptake decreases with higher storage temperatures as reflected by the side scatter (SSC) (Figure 5B), the number of viable cells (Ax-PI-) increases. After 24 h of incubation with 300 µg/mL total iron, the relative number of cells which are negative for both stainings decreases significantly (*p* < 0.05 or smaller) in a temperature-dependent manner.

Using a hexamethylindodicarbocyanine iodide dye (DiIC_1_), cells with intact mitochondrial membrane potentials were detected. The results of this staining were in concordance with the Ax/PI staining, as the percentage of viable (DiIC_1_+) cells decreased depending on total iron concentration. Increasing the storage temperature of the particles resulted in increasing numbers of viable cells at high iron concentrations (Figure 5C).

During cell death, cellular nuclei are often degraded. We therefore additionally analysed cellular DNA in terms of fragmentation (sub-G1 DNA) using propidium iodide (PI) staining in the presence of the detergent Triton X-100, which lyses cellular membranes and enables PI to freely intercalate into the DNA. In concordance with previous results, the percentage of cells with degraded DNA increased depending on the particle concentration; again, this effect was reduced by higher storage temperatures of the particles (Figure 5D).

**Figure 6:**
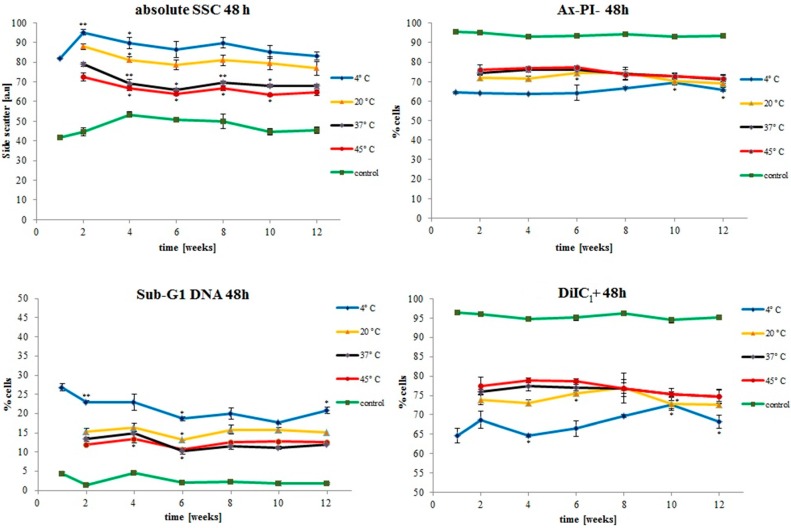
Time dependence of SEON^LA–BSA^ biocompatibility on Jurkat cells depending on storage temperature and storage time. The lines represent untreated control (green line and dots), particles stored at 4 °C (**blue**), 20 °C (**yellow**), 37 °C (**black**) and 45 °C (**red**) at concentrations of 300 µg/mL total iron. The absolute side scatter (SSC) is not normalized to the untreated controls. All experiments were performed in triplicates. The asterisks mark significant differences in comparison to the previous values. The significance levels were determined using an unpaired *t*-test (* *p* < 0.05; ** *p* < 0.005).

The impact of nanoparticle storage temperature on aforementioned parameters over the whole experimental period of twelve weeks is displayed in Figure 6. The concentrations used for all these graphs are 300 µg/mL total iron. As is evident from the time graphs, the main effect on biocompatibility of SEON^LA–BSA^ occurs at the beginning of storage at the different temperatures. After that the differences between the particles stored at the different temperatures do not change greatly over the rest of the observation period.

### 2.3. Discussion

The colloidal stability of SEON^LA–BSA^ was not affected by the given storage conditions as the hydrodynamic size of the clusters did not change significantly after the observed time period. It should be noted, however, that some single values differed significantly from the original value, which we attribute to normal deviations of dynamic light scattering between the single measurements. Iron oxide suspensions have shown to be colloidally stable during storage at elevated temperatures before [24], however, previous studies did not include higher temperatures or potentially fragile coatings like albumin. The zeta potential of around −30 mV is consistent with previous findings [21]. This is indicative of the high electrostatic stability of the particles during storage at the given conditions. Surface adsorption of ions does also seemingly not occur during storage. Previous studies on albumin coronas showed a great temperature-dependence of the shape and stability of such protein coronas on nanoparticles [20]. However, in case of SEON^LA–BSA^ we proposed earlier that the protein shell is not randomly arranged around the clusters by electrostatic interaction, but chemisorbed using lauric acid as anchoring groups on the iron oxide surfaces [21]. The high, temperature-independent colloidal stability of these particles under these conditions can be further supportive of that theory.

The different temperatures did not affect crystal growth of the iron oxide cores significantly. Iron oxide nanoparticles have been reported to be sensitive to Ostwald ripening [18], however, coatings by organic molecules can passivate the iron oxide surface [25] against the release of iron ions and therefore hinder this process. We also did not observe any chemical changes of the coating moieties in the routinely performed FT-IR measurements. This good stability of albumin at the given conditions seems surprising on the first look. However, fatty acids have been shown to stabilize albumin solutions, enabling heating up to 60 °C [26] without aggregation or denaturation taking place.

The suspension underwent a macroscopic colour change over time. The oxidation of magnetite to maghemite in contact with oxygen following the reaction:

(1) $${\text{Fe}}_{3}{\text{O}}_{4}+2{\text{H}}^{+}\;\overset{\;}{\to }\;\text{γ}-{\text{Fe}}_{2}{\text{O}}_{3}+\;{\text{H}}_{2}\text{O}+\;{\text{Fe}}^{2+}$$

is a well-known effect [27,28]. Salazar *et al.* have demonstrated that the surface of magnetite nanoparticles is very quickly oxidized, whereas deeper oxidation occurs over time and depending on the temperature [29]. They also observed a similar effect on the saturation magnetization, which correlated with the magnetite/maghemite ratio. Nanoparticles of the two magnetic iron oxides magnetite and maghemite are not so easy to discriminate in XRD, because the two spectra are very similar [30]. For small particles with crystal defects, the crystal diffraction of the X-ray beam can be inhomogeneous and thus cause broadening of the peaks [27]. However, our results confirm the oxidation of Fe_3_O_4_ and explain the trend of the M_S_ values. Along with oxidation of the iron oxide cores the saturation magnetisation of the suspension decreases in a time- and temperature dependent manner during storage. Again the main effect occurs during the first four weeks. As magnetite possesses higher M_S_ than maghemite [16], this change in M_S_ could be related to oxidation processes involving the formation of the latter. Compared to other oxidative processes in the literature [28,29] this oxidation is rather slow, which could be due to passivation of the surface by the organic coating. After twelve weeks of storage, the M_S_ are still not fully equalised, therefore we conclude that oxidation is not yet quantitative. Long-term experiments (up to 1 year and above) shall be the topic of our future research.

Biocompatibility assays showed a decrease in toxicity of SEON^LA^^–^^BSA^ with increasing storage temperature and time. Nevertheless it should be noted that toxic effects only occurred at very high concentrations of 200 µg/mL and above. This effect goes along with aforementioned decrease of M_S_ which we proved to be related to oxidative processes, so it is likely that it is also related to oxidation of the iron oxide cores. At the same time, the uptake of particles was reduced accordingly, so the differences in the toxicities could be deriving just from reduced cellular uptake. Toxicity of particles has been frequently shown to be dependent on their hydrodynamic size and surface charge [12,13]. Comparative studies of magnetite- and maghemite-based particles have shown different toxicities and cellular uptakes before [14,15]. In many cases, however, particle size charges and surface coatings of the investigated particles are not always identical and thus the differences cannot be directly attributed to core oxidation alone. In this study we proved that both size and zeta potential stay constant regardless of the oxidation of the cores, so in this case the observed effects could be related to each other directly. As the oxidation of magnetite to maghemite involves the deposition of iron (II), which is one potential cause of oxidative stress after cellular uptake of SPIONs and their decomposition in lysosomes [31,32], this could be one of the reasons behind the observed effects. The exact uptake mechanism of SEON^LA^^–^^BSA^ has not been clarified. Other studies suggest that the uptake of SPIONs occurs mainly via endocytosis [32,33]. Apparently, the core of the particles could play a certain role in the cellular uptake as well, reducing the uptake and thereby decreasing toxic effects as the particles get oxidised.

## 3. Experimental Section

### 3.1. Materials and Chemicals

Iron (II) chloride tetrahydrate (FeCl_2_·4H_2_O), hydroxylammonium chloride and bovine serum albumin (BSA), were purchased from Merck (Darmstadt, Germany). Iron (III) chloride hexahydrate (FeCl_3_·6H_2_O), dialysis tubes (Spectrapor 6, MWCO 8 kDa), ammonium chloride, formic acid, hydrochloric acid 25%, and ammonia solution 25% were supplied by Roth (Karlsruhe, Germany). Propidium iodide (PI), sodium citrate, triton X-100, ammonium formate, lauric acid and acetone were purchased from Sigma-Aldrich (St Louis, MO, USA). Sulfosalicylic acid solution 20% was bought from Applichem (Darmstadt, Germany). Ringer’s solution was bought from Baxter Healthcare (Zurich, Switzerland). Falcon ultrafiltration tubes (MWCO 100 kDa) were purchased from Sartorius (Gottingen, Germany). Annexin A5-Fluorescein isothiocyanate (Ax), Hoechst 33342 (Hoechst) and DiIC_1_(5) (DiI) were purchased from Thermo Fisher Scientific (Waltham, MA, USA).

European Pharmacopoeia quality transparent glass vials (type 1), rubber closures and aluminium crimp caps were purchased from Zscheile and Klinger GmbH, Hamburg, Germany.

### 3.2. Cells and Culture Conditions

Jurkat cells, a non-adherent human T cell leukemia cell line (DSMZ ACC 282), were cultured in RPMI 1640 medium supplemented with 10% fetal calf serum (FCS) and 2 mM l-glutamine (both from Thermo Fisher Scientific, Waltham, MA, USA) under standard cell culture conditions at 37 °C and 5% CO_2_.

### 3.3. Synthesis and Storage of SEON^LA^^–^^BSA^ Particles

Superparamagnetic iron oxide nanoparticles were synthetized as described previously [21]. Briefly, iron oxide nanoparticles were synthetized by co-precipitation from a mixture of iron (II) chloride tetrahydrate and iron (III) chloride hexahydrate (molar ratio Fe^3+^:Fe^2+^ = 2) by addition of NH_3_ at 80 °C under Ar atmosphere and colloidally stabilised by *in situ* addition of lauric acid at 90 °C. After purification by dialysis, the artificial protein corona was formed by mixing the suspension with 20% bovine serum albumin (BSA) solution under vigorous stirring. The particles were then purified by centrifugal ultrafiltration (MWCO 100 kDa) and subsequently sterile filtered through a 0.22 µm filter membrane portioned into sterile glass vials and sealed under sterile conditions. The level of filling was such that half of the vial volume (V_Vial_ = 10 mL) was filled with nanoparticle suspension, the other half consisted of ambient air. The total iron concentration was determined using a previously described ultraviolet (UV) spectroscopy method [34]. Twenty vials, each containing 5 mL of SEON^LA^^–^^BSA^, were then stored at 4 ± 1, 20 ± 1, 37 ± 1, or 45 ± 1 °C over the course of twelve weeks. They were stored in the dark in order to minimise effects from light exposure. One vial was handled under Ar atmosphere directly after synthesis and used to obtain the XRD spectra at week 0.

### 3.4. Hydrodynamic Size and Zeta Potential Determination

Hydrodynamic size measurements were performed using a Nanophox (Sympatec, Clausthal-Zellerfeld, Germany). SEON^LA–BSA^ particles were diluted to 25 µg/mL total iron with ultrapure water and measured in triplicates at 25 °C in cross-correlation mode.

The zeta potential was determined using a NICOMP 380ZLS (Nicomp, Port Richey, FL, USA) using the same dilution as for the size measurements. The zeta potential was calculated using the Smoluchowski equation. All measurements were performed at 25 °C in triplicates.

### 3.5. Statistical Analysis

Statistical analysis was performed using either a two-tailed Student’s *t*-test in Microsoft Excel. Values of *p* < 0.05 were considered as statistically significant. Paired tests were used for the physicochemical measurements, unpaired tests were used for the biological measurements. The nomenclature in all figures is: * *p* < 0.05; ** *p* < 0.005; *** *p* < 0.0005.

### 3.6. Fourier Transform Infrared (FT-IR) Measurements

Five hundred µL of the respective particles were lyophilized overnight. Spectra of the respective samples were then taken with a BRUKER Alpha FT-IR spectrometer (Bruker, Billerica, MA, USA) operated in attenuated total reflection mode from 4000 to 400 cm^−1^ using a step size of 0.5 cm^−1^.

### 3.7. Transmission Electron Microscopy (TEM)

For sample preparation the respective particle suspensions were diluted 1:200 in ultrapure water. Samples were then prepared by drying 10 μL of diluted nanoparticle suspension on a carbon-coated Athene S147-2 copper grid (Plano, Wetzlar, Germany) under constant airflow. Pictures were taken with a CM 300 UltraTWIN (Philips, Eindhoven, The Netherlands) operated at an acceleration voltage of 300 kV. Images were obtained with a CCD camera; for determination of the size distribution of the iron oxide cores the software ImageJ 1.47 was used.

### 3.8. X-ray Diffraction (XRD)

Atomic structure of crystalline substances can be determined through X-ray diffraction (XRD). This study used a X’PERT MPD Pro (PANalaytical, Almelo, NL, USA) XRD device. X-rays fall on the sample with an angle θ and are diffracted with different intensities due to different crystal plane orientations. Samples were prepared by lyophilisation directly after synthesis and handling under Ar atmosphere and after eight weeks of storage at the different temperatures.

### 3.9. Vibrating Sample Magnetometry (VSM)

To enable a magnetic characterization of the samples a vibrating sample magnetometer, the 7407 (Lake Shore, Westerville, OH, USA) was used. The samples were identically prepared and the magnetization curves were measured for increasing and decreasing external magnetic fields. The saturation magnetizations of the ferrofluids were determined at high external magnetic fields of up to 1000 kA/m. We measured the samples at given time points over the course of twelve weeks in order to investigate the time-dependent correlation between storage temperature and magnetic susceptibility of the ferrofluids.

### 3.10. Flow Cytometry Measurements

Before every experiment, the viability and cell count of the Jurkat cells was determined using a Muse Cell Analyzer (Merck Millipore, Darmstadt, Germany). If the cell viability exceeded 90%, we proceeded with further experiments. The cells were adjusted to a density of 2 × 10^5^ cells per mL in cell culture media. The cell suspensions were seeded into 48 well plates (Greiner Bio-One, Frickenhausen, Germany) with the respective amount of particles (final concentrations were ranging from 50, 100, 200 and 300 µg/mL total iron; final volume 1 mL each) and incubated at 37 °C. Untreated cells served as controls. After 24 and 48 h, 50 μL aliquots of the cell suspensions were taken and stained in order to perform analyses of phosphatidylserine exposure, plasma membrane integrity and mitochondrial membrane potential as previously reported by Munoz *et al.* [23].

Briefly, 250 μL of a mixture of PI 20 μg/mL, DiIC_1_ 10 nM, Hoechst 1 and 0.5 μg/mL AxA5-Fluorescein isothiocyanate in Ringer’s solution were added to each of the aliquots, and incubated for 20 min at 4 °C.

The tubes were subsequently measured with a Gallios flow cytometer (Beckman Coulter, Fullerton, CA, USA) for 60 s. Electronic compensation was used to eliminate bleed through fluorescence. The forward scatter (FSC) and side scatter (SSC) were considered for morphological analysis of the cells. Data analysis was performed with the Kaluza software version 2.0 (Beckman Coulter, Fullerton, CA, USA).

Cell cycle and DNA degradation were examined by propidium iodide-triton (PIT) staining [35]. Briefly, 400 μL of a solution containing 0.1% sodium citrate, 0.1% Triton X-100 and 50 μg/mL PI were added to another 50 μL aliquot of cells, incubated overnight at 4 °C in the dark and nuclear fluorescence was measured in flow cytometry.

## 4. Conclusions

From our results we conclude that low storage temperatures can slow storage-related changes in the physicochemical properties and biocompatibility of SEON^LA–BSA^ over the course of several weeks. It therefore seems evident that 4 °C is the optimum storage temperature for this kind of iron oxide nanoparticle. Although it should be noted that in the chosen range of conditions, changing the storage temperature had only moderate influence, the main effects in the observation timeframe were related to a significant decrease of saturation magnetization and a reduction in cellular uptake. Both effects correlated with an oxidation of the iron oxide particle cores from Magnetite to Maghemite. Colloidal stability and chemical composition of the shell were not affected at the given conditions.

Additionally, the results allow a direct correlation between oxidation of the cores and toxicity of iron oxide nanoparticles.
